# *In vitro* screening methods for parasites: the wMicroTracker & the WormAssay

**DOI:** 10.17912/micropub.biology.000279

**Published:** 2020-07-20

**Authors:** Emma Gunderson, Christina Bulman, Mona Luo, Judy Sakanari

**Affiliations:** 1 University of California, San Francisco

**Figure 1:**
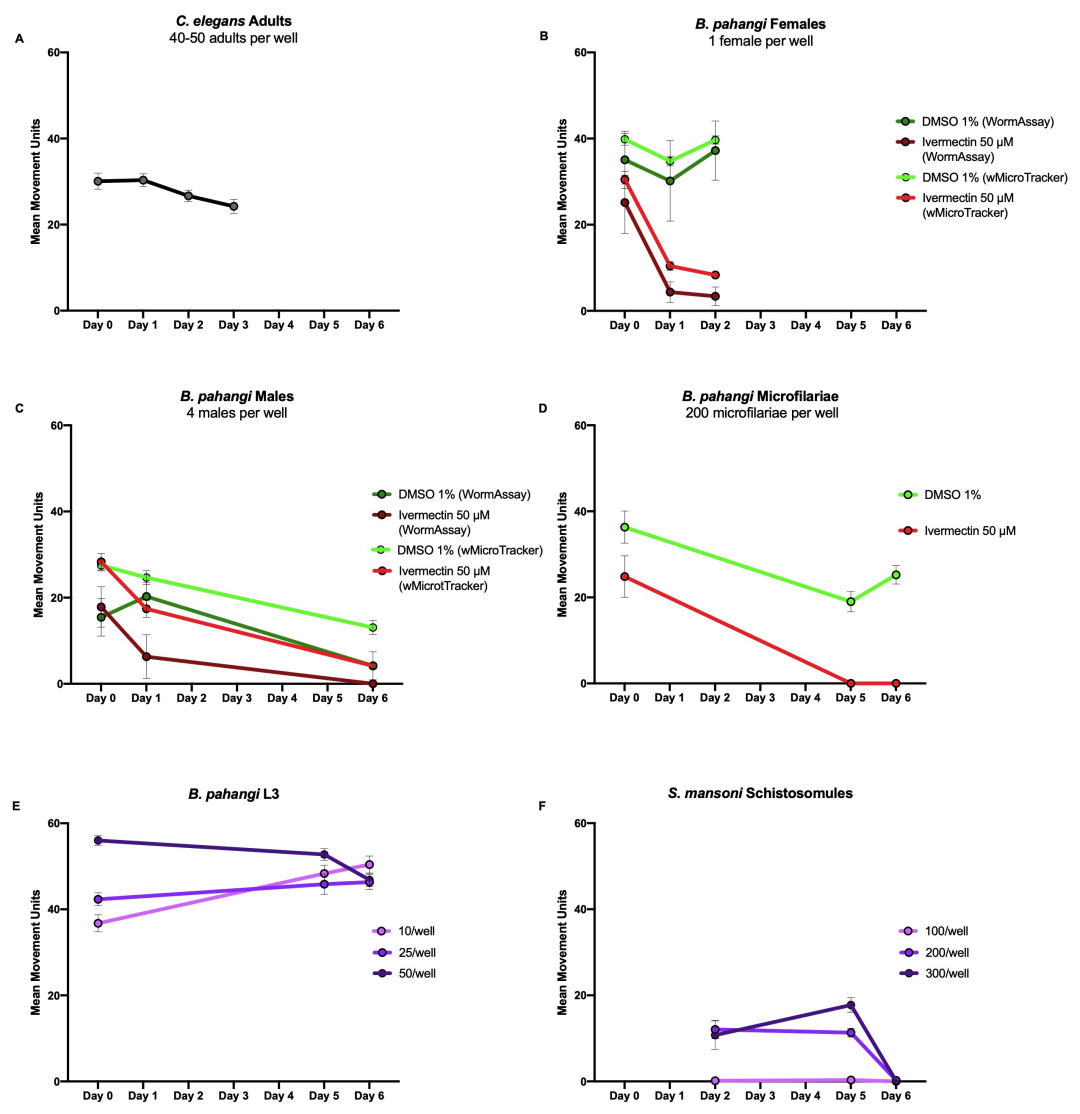
Results of motility screens of C. elegans adults (A) in the wMicroTracker, *B*. *pahangi* females (B) & *B. pahangi* males (C) in both the WormAssay and the wMicroTracker, and *B. pahangi* microfilariae (D) , *B. pahangi* L3 (E) and *S. mansoni* schistosomules (F) in the wMicroTracker. Data are presented as mean ± 95% CI.

## Description

The purpose of this study was to determine the utility of the wMicroTracker as a screening platform to assess the motility of various parasites. We tested three species of parasites: the adult and larval stages of the filarial nematode *Brugia pahangi*, the schistosomula stage of the trematode *Schistosoma mansoni*, and the epimastigote stage of the protozoan parasite *Trypanosoma cruzi*.We optimized the assay for the number of parasites per well, plate type and media volume using the wMicroTracker and compared those readouts to readouts from the WormAssay (Marcellino *et al.* 2012) when possible. The WormAssay has been used in phenotypic drug screens to identify new compounds for the treatment of lymphatic filariasis, onchocerciasis and schistosomiasis (Storey *et al.* 2014; Bulman *et al.* 2015; Weeks *et al.* 2018; Tyagi *et al.* 2019). The original WormAssay was developed by Marcellino *et al.* 2012 and was subsequently modified to the “Worminator” by Storey *et al.* 2014 to observe smaller worms with an inverted microscope. wMicroTracker (InVivo Biosystems) protocols optimized for *C. elegans* adults (1 mm in length by 80 µm in width, highly motile) were used to optimize parasite assays based on the size and motility of each of the parasite species as compared to *C. elegans* adults.

To use the wMicroTracker, two factors need to be considered: the size of the parasite of interest and how active they are. The wMicroTracker detects movement when an organism crosses the stationary LED beam at the center of the well. Hence, parasites that do not travel throughout the well should be assayed in a U-bottom plate to ensure movement is detected. For parasites that do travel throughout the well, a flat bottom plate is sufficient. To determine the number of parasites to use per well, we compared their size to that of *C. elegans* adults. Following the InVivo Biosystems protocol (https://invivobiosystems.com/wp-content/uploads/2018/12/Protocol_toxicity-in-c-elegans_122018.pdf), *C. elegans* adults were screened in the wMicroTracker with 40-50 adults per well in 100 µL of M9 buffer (Stiernagle 2006). This produced results in the range of 25-35 mean movement units per well (Figure 1A). To optimize screening methods in the wMicroTracker for other parasites, the size and motility of parasites of interest were compared to *C. elegans* adults to ensure control wells gave mean movement units around this range.

*B. pahangi* females (34.7 mm in length by 139 µm in width (Mutafchiev *et al.* 2014), highly motile) were screened with one worm per well in 500 µL of RPMI in a 24-well flat bottom plate with 8-9 replicate wells. *B. pahangi* males (18.0 mm in length by 77 µm in width (Mutafchiev *et al.* 2014), high motility) were screened with four worms per well in 500 µL of RPMI in a 24-well flat bottom plate with 4-7 replicate wells. *B. pahangi* females and males were assessed in both screening platforms. Both platforms showed similar motility profiles in response to 50 µM ivermectin or 1% DMSO as a negative control (Figure 1B & C).

*B. pahangi* microfilariae (mf) (177-230 µm in length by 5-7 µm in width (CDC, Lymphatic Filariasis), moderately motile) were screened in the wMicroTracker with 200 mf per well in 100 µL RPMI in both a 96-well U-bottom plate (Figure 1D) and a 96-well half-volume flat-bottom plate (data not shown). Mf were screened with 18 replicate wells per treatment where the positive control was 50 µM ivermectin and the negative control was 1% DMSO. The 96-well half-volume flat-bottom plate was not able to detect mf movement.

*B. pahangi* third-larval stage (L3) (1-2 mm in length by 26 µm in width (Mutafchiev *et al.* 2014), highly motile) were screened in the wMicroTracker with 10, 25, and 50 L3 per well in 200 µL RPMI in a 96-well U-bottom plate with 3 replicate wells per condition (Figure 1E). All conditions showed similar motility profiles and produced reliable data.

*Schistosoma mansoni* schistosomules (110 µm in length by 18 µm in width (Samuelson *et al.* 1980), not very motile) were screened in the wMicroTracker with 100, 200, and 300 schistosomules per well in 100 µL RPMI in a 96-well U-bottom plate with 8 replicate wells per condition (Figure 1F). Both 200 and 300 schistosomules per well showed generally similar motility profiles and gave reproducible results.

*Trypanosoma cruzi* epimastigotes (25.6 µm in length by 1.9 µm in width (Gonçalves *et al.* 2018), moderately motile) were also screened in the wMicroTracker with 10,000, 50,000, and 100,000 epimastigotes per well in 100 µL DMEM in a 96-well U-bottom plate with 6 replicate wells per condition (data not shown). Motility was not detected in the wMicroTracker. Based on these data, it is not known if more than 100,000 parasites per well would allow for the wMicroTracker to detect motility of *T. cruzi* epimastigotes.

Based on the results of our screens with various parasites using the wMicroTracker and the WormAssay, it is recommended that the WormAssay be used to screen parasites greater than 10 mm in length and that the wMicroTracker and the modified WormAssay, the “Worminator” (Storey *et al.* 2014), be used to screen parasites less than 1 mm in length. When screening parasites in the wMicroTracker that do not travel throughout the well (e.g. schistosomules, *B. pahangi* mf, *B. pahangi* L3) it is recommended to employ a 96-well U-bottom plate to ensure the parasites do not settle at the sides of the well and that they are able to cross the stationary LED beam at the center of the well. When screening parasites that are larger than *C. elegans* adults in the wMicroTracker, use fewer parasites per well and when screening parasites in the wMicroTracker that are smaller than *C. elegans* adults, use more parasites per well. The aim is to detect mean movement units around 20-40 per well. We believe that these methods will help the research community develop better screening methods and shorten the time to optimize a screening platform.

## Reagents

RPMI: Thermo Fisher Scientific (Catalog #: 22400089)

DMEM: Sigma-Aldrich (Catalog #: D5796)

Ivermectin: Sigma-Aldrich (Catalog #: I8898)

DMSO: Fisher Scientific (Catalog #: BP231-100)
